# The Maudsley Anorexia Nervosa Treatment for Adults (MANTRA): a feasibility case series of an integrated group based approach

**DOI:** 10.1186/s40337-021-00424-6

**Published:** 2021-06-15

**Authors:** Helen Startup, Mary Franklin-Smith, William Barber, Nicola Gilbert, Yael Brown, Danielle Glennon, Akira Fukutomi, Ulrike Schmidt

**Affiliations:** 1 Sussex Partnership NHS Foundation Trust, Sussex Eating Disorders Service, Brighton, UK; 2 Leeds Partnership NHS Foundation Trust, CONNECT: The West Yorkshire Adult Eating Disorder Service, Leeds, UK; 3 South London and Maudsley NHS Foundation Trust, Eating Disorders Outpatient Service, London, UK; 4 Department of Psychological Medicine, King’s College London, Institute of Psychiatry, London, UK

**Keywords:** Anorexia nervosa, Eating disorders, Group treatment, Emotion regulation

## Abstract

**Background:**

Individuals with Anorexia Nervosa (AN) typically struggle in social and emotional contexts. An Integrated Group Based approach for the delivery of MANTRA - The Maudsley Anorexia Nervosa Treatment for Adults – extends current NICE recommended therapy by augmenting treatment with opportunities for experiential practice in a group context. A feasibility case series, delivered across three NHS community services is presented.

**Methods:**

The design was a case series of four Integrated Group MANTRA treatments delivered across three NHS sites (*N* = 29). Feasibility data of: retention, acceptability and effectiveness; alongside the qualitative capture of participant experiences of treatment is presented.

**Results:**

Primary outcomes suggest treatment acceptability. Participants committed to treatment with only 2 dropouts. There was significant change with medium effect sizes for eating disorder cognitions and symptoms (as measured by the global score on EDEQ) and BMI. Core themes emerging from qualitative analysis captured the value of the relational aspect of the treatment, the incorporation of experiential methods, and the opportunity to draw on the support of the group members to reduce shame and stigma.

**Conclusions:**

An Integrated Group based MANTRA approach is a feasible and effective alternative intervention for community Eating Disorder services.

## Plain English summary

Treatments for Anorexia Nervosa (AN) are somewhat effective, but there is room for improvement. A core struggle for individuals with Anorexia Nervosa is managing emotions especially in a social context. One of the leading treatments for AN - MANTRA – was adapted to be delivered in a group to provide opportunities for individuals to practice experiencing and managing emotions amongst others. We hoped that being in a group could help tackle the shame and isolation that many people with AN endure. Patients seemed to find value in this approach and there are early signs that it may support people on their journey of recovery from Anorexia Nervosa.

## Background

Anorexia Nervosa (AN) characterised by restricted eating, driven by intense fear, in the context of weight, shape and eating concerns [1] has the highest mortality rates of any psychiatric disorder [2, 3]. Incidence is rising; both community service demand and inpatient admissions have increased [NHS Digital, 2017 (digital.nhs.uk)]. AN is not simply a disorder of eating - 60% of individuals have significant personality disorder features (such as emotional dysregulation) and many have anxiety, depression, OCD and other Axis 1 presentations [4, 5]. This routine occurrence of co-morbid difficulties indicates an evidence-based, modularised approach to conceptualisation and treatment.

The Maudsley Anorexia Nervosa Treatment for Adults (MANTRA) offers this [6]. It is NICE recommended and used across UK services and internationally [7]. MANTRA is as effective as the other NICE recommended treatments at improving AN outcomes [8–12]. A Cognitive-Interpersonal Maintenance Model specifies 4 domains of biological and psychological predisposing and maintaining factors: 1) the emotional and social mind, 2) identity, 3) thinking styles, and 4) relationships [13, 14] which map onto intervention targets. MANTRA is a manualised modularised flexible and effective treatment for AN; well liked by patients and therapists [15, 16]. However, outcomes for all 3 leading AN treatments lag behind outcomes for other Axis 1 Presentations [17]. Opportunities are potentially being lost in translation of the known maintenance factors at treatment delivery.

A core MANTRA maintenance loop involves the emotional and social mind. Individuals with AN can struggle with emotional processing, particularly in interpersonal contexts [18, 19]. Difficulties include; identification of emotions, emotional tolerance and integration of emotional material into the self and sense of self in the social world [19–23]. Unfortunately, the starved state achieves emotional bluntness effectively [24], and high levels of worry and rumination can further suppress emotion and worsen ED symptoms [25]. FMRI studies confirm this pattern; individuals with AN who exhibit emotional suppression or distancing show high rumination and worse weight gain outcomes [26]. Individuals are drawn to AN to cope with limited ‘real world’ opportunities to update this coping repertoire [19].

A body of work has focused on enhancing emotional processing in the treatment of those with AN (cf. [27]), however, treatments to date underestimate the importance of direct, meaningful interpersonal exposure to maximise change in social and emotional domains. Opportunities to support the emotional regulation of others, as well as receiving emotional support can target affective and interpersonal deficits [18, 28–30]. Furthermore, the Window of Emotional Tolerance (WoT) for those with AN is narrow; intellectualised - or ‘paper and pencil’ type approaches to therapy can enable patients to remain ‘safe’ yet emotionally ‘cut off’ blocking social and emotional processing [31]. Chair work, for example, has shown promise in fostering compassion in relation to the Anorexic Voice [32], and imagery techniques can augment classic CBT when challenging the ‘restrictive mode’ in AN [33]. Creative approaches with experiential foci, such as drama therapy methods [34, 35], chair work [36, 37], imagery work [38] and play [39] stretch an individual’s WoT encouraging new emotional experiences to update old schemata.

Treatment delivery that maximally targets the Emotional and Social Mind mechanism within the MANTRA treatment is important and may be achieved by offering MANTRA within an integrated, group format. The modular arrangement of MANTRA lends itself to a group format whilst more idiosyncratic elements can be delivered within individual sessions. This combination of individual and group formats works well for the Emotional and Social Mind Integrated Bulimia Nervosa treatment [40] and is advocated in the broader psychotherapy literature [41]. The group format provides a supportive live arena for giving and receiving emotional regulation experiences (via attunement, supported social problem solving, safe disclosure of behaviours deemed shameful, as well as self- and other-directed compassion) and for stretching the WoT via the incorporation of in situ experiential methods (such as imagery and chair and role work) which are more likely to enhance emotional connectedness and provide opportunity to update schemata (cf. [36]). It has been highlighted that the group setting serves as an ‘interpersonal laboratory’ to practice interpersonal skills [42]. Dynamics such as unhelpful competition between group members, sometimes highlighted as a reason against group treatments for AN, can be explored, alongside the elements of group facilitation required to navigate this and turn it to therapeutic use. Individual sessions have a practical focus and foster alliance building. Dips in motivation are discussed, idiosyncratic aspects of an individual’s formulation can be addressed and ED relevant goals negotiated and monitored. They provide space to enable any areas that might inhibit the individual to make maximum gain from the group programme to be addressed, therefore guarding against drop out.

This paper describes MANTRA treatment delivery translated into an integrated group-based model, drawing on theories of emotion regulation in context [28], dramatherapy methods [34, 35] and experiential treatments such as schema therapy [36]. Interventions are ‘lifted’ off the page, encouraging individuals from staying safe yet ‘cut off ‘and providing opportunities for social and emotional learning and to directly target schemata around shame and isolation. Retention, acceptability and preliminary treatment outcome data is presented regarding this new integrated group-based treatment as delivered across three NHS sites in the UK. Outcomes highlight the potential benefits of an integrated group-based MANTRA approach for patients with Anorexia Nervosa.

## Method

### Ethical considerations

The UK National Health Service National Research Ethics Service guidance [43] established that this study did not require ethical approval as outcomes were collected as part of routine clinical practice. All participants were aware that their data could be used for evaluation purposes and that all data would be fully anonymised assuring no breaches of confidentiality.

### Design

An uncontrolled case series design was used to examine the evidence for an integrated group-based MANTRA intervention. We refer to the treatment as an ‘integrated’ approach because of the combination of both group and individual sessions. Participants were recruited from three specialist adult eating disorder services between September 2018 to March 2020. Outcomes were reported at assessment, prior to treatment, upon completion of treatment and, for two of the sites, (South London and Sussex), at 6-month follow up. In addition, qualitative group feedback, using semi-structured post intervention questions, was collected from participants and therapists post intervention.

### Participants

Participants were recruited from 3 NHS Outpatients ED service sites across England: South London and Maudsley Foundation Trust (Site 1), Sussex Partnership Foundation Trust (Site 2), Leeds and York Partnership Foundation Trusts (Site 3). Consecutive referrals were offered the group if they were; (a) > 18 yrs., and (b) had a DSM-IV diagnosis of anorexia nervosa or Other Specified Feeding Disorder (OSFED), with a body mass index (BMI) < 18.5 kg/m^2^. Patients were excluded if they had life-threatening AN requiring intensive treatment, or had insufficient knowledge of English language to engage in the treatment, intellectual disability, severe mental or physical illness needing treatment in its own right (e.g. psychosis), substance dependency or pregnancy.

### Outcome measures

Clinical assessments (BMI and the Eating Disorder Examination Questionnaire) took place before and after the group and at 6 months follow up for sites 1 and 2. Group evaluation feedback forms were completed at the final group session.

#### Body mass index (BMI)

Patient BMI was recorded at each time point.

#### Eating disorder examination questionnaire (EDE-Q 6.0, [44, 45])

The EDE-Q is a 28-question measure of severity for eating disorders. Items are a combination of open-ended questions requesting frequency of behaviours as well as scoring responses from 0 (no days) to 6 (everyday). Responses are summed into a global score for eating disorder psychopathology by averaging the four EDE-Q subscales: Restraint, eating concern, shape concern and weight concern.

### Group evaluation forms

Self-report questionnaires were used to evaluate participants’ experience of group-based MANTRA. These were standard service related measures, largely open-ended and focused on the participant experience of the intervention.

### Intervention

Group-based MANTRA has been adapted from the standard MANTRA manual [6] with the goal of augmenting change in the social and emotional domain through the use of group processes and live experiential methods [46]. Of note the MANTRA manual is not designed as a ‘paper and pencil’ workbook, it is a useful guide to supplement collaborative clinical work. The experiential interventions within this integrated group treatment are in line with theoretical goals underpinning the standard manual, but are of particular relevance and utility to a group setting.

### Format of the intervention

Integrated Group MANTRA combines a group delivery format with a number of individual sessions. The group aspect is delivered over twenty, weekly, 90-min group sessions. Two group facilitators each take half of the group members for their individual sessions. Patients are offered 2–8 individual therapy sessions in addition to the group programme, at least two of these being prior to the group starting. The aim is for a group size of 8 to 12 patients. Group facilitators adopt a motivational, curious stance. The group schedule is roughly organised as in Table 1. The session format involves: group check-in, the main event or session topic, and a group check-out, with homework usually from the MANTRA workbook [6] and stemming from the topic of the session.

**Table 1:** Key features of the integrated group MANTRA programme

Module (workbook chapter)		Number of sessions	Content
5	Formulation	1–4	1:1 vicious flower formulation, preparing for working in a group, working with loved ones in their support network and SMART goal setting.
7	Social and Emotional Mind	6	Understanding of emotions/feelings, range of emotions, feelings and behaviours towards loved ones, externalising the inner critic, reflecting and responding to emotions.
8	Thinking Styles	4	Identifying thinking style, how thinking styles perpetuates AN, and alternative thinking styles.
9	Identity	4–6	Sense of identity with and without ED, personal values and interests/hobbies, practicing the healthy flourishing self, challenging ED rules.
4	Nutrition	3–4	Each module has an attached nutrition model to link course learning and diet. Module 1) Link between food and mood; Module 2) how diet impacts/influences thinking style; Module 3) how their sense of self is affected by nutritional needs. There is one additional session which focuses on relapse prevention and nutritional needs post discharge.

### Individual sessions

Patients have at least two individual sessions prior to the group commencing. If indicated a further two can be spaced out over the course of the intervention. Each individual session is an hour long and practical rather than experiential in foci. The goal of these sessions is: 1) to introduce the manual, to establish baseline motivation and enhance this by drawing on exercises from relevant early MANTRA workbook sessions, including formulation 2) to psycho-educate in regard weight, nutrition and links to the putative maintenance factors and to set initial weight and nutrition targets, 3) to provide time for questions and to work through any worries or practicalities around joining the group. Ongoing individual sessions are used flexibly alongside the group programme. Addition individual sessions were used to problem solve idiosyncratic concerns that arose as the treatment progressed that were not fully addressed in the group. Examples included provision of psycho-education and guidance around managing binge/purge cycles, discussing interpersonal worries triggered outside of the group, or for one individual considering the role of increased flashbacks as weight increased.

### Management of Risk

Prior to each group session patients were weighed by a clinician; they arrived 15 mins ahead of the group so that this could be done without impacting on group time. Where possible their allocated therapist was also the person who weighed them and weighing took place in a clinic room away from other group members. Time was allowed for a brief check-in around weight and nutrition goals, as well as exploring the emotional impact of any weight change directly within the group setting. If further specific clinical support was needed, such as time to discuss a boost to nutrition goals or involvement of a family member, additional individual sessions would be scheduled (in line with the protocol). Where additional stepped up care was indicated this was managed in the usual way by the service.

### Key apparatus

Communicube. This is used to support check-ins and outs (www.communicube.co.uk). A Communicube is a clear structure of shelves with 5 levels, for the purposes of the group we tend to work with these levels as symbolic of levels of conscious awareness, but they can also be used to think about’ levels’ present within our bodies, our families and the group itself. Group participants are invited to select an object or card (objects were gathered via charity shops as well as an email request around the departments to bring in any small objects, toys, pictorial cards etc.) to reflect their emotional state and the level of awareness they had of their current emotional world. Common emotional themes often fed into the main topic of discussion. At the check-out patients are invited again to reflect on the session using the Communicube. Reflections do not need to be verbalised and can be represented using an object, sometimes other group members put voice to the feeling state.

### Session focus and delivery methods

The main event changes by module and session. Each session aims to target one of the putative maintenance factors and there are a range of delivery methods for achieving this. Some of these methods are drawn directly from the MANTRA manual and some were specific in delivery to the group format. Therapists flexibility choose from a range of different ways of working, some of which are listed in Table 1 and full details are in Franklin-Smith (2019).

### Treatment fidelity

All therapists had been trained and were experienced in delivering individual MANTRA, training in the group model was provided by one of the authors, MFS. The three sites met regularly to assure adherence to the therapist manual [46] for the group intervention as well as to share ongoing practice and supervisory reflections and learnings.

### Data analysis

Quantitative data were analysed using IBM SPSS Statistics Version 26. Demographic data is presented as means, standard deviation and frequency where appropriate. Paired sample t-tests were used to compare means at assessment with post-group for BMI, and because of insufficient EDEQ data at assessment, means were compared for this variable at baseline and post-group. Effect sizes are reported using Pearson’s r correlation coefficients. Follow up data were available for two participating sites and therefore an additional repeated measures one-way ANOVA was run comparing BMI and timepoint.

#### Qualitative

The open-ended questions on the group evaluation forms were analysed using framework analysis methodology [47]. Authors HS, NG and WB familiarised themselves with the data and through regular discussion developed a framework consisting of five overarching themes from issues originating in the familiarisation stage, as well as a priori concerns about the adaptation of MANTRA for a group setting. Data were then indexed onto this framework and summarised, following which authors HS, NG, and WB collectively mapped and interpreted the data, discussing alternative explanations and inconsistent findings.

## Results

### Participants

**Fig. 1:**
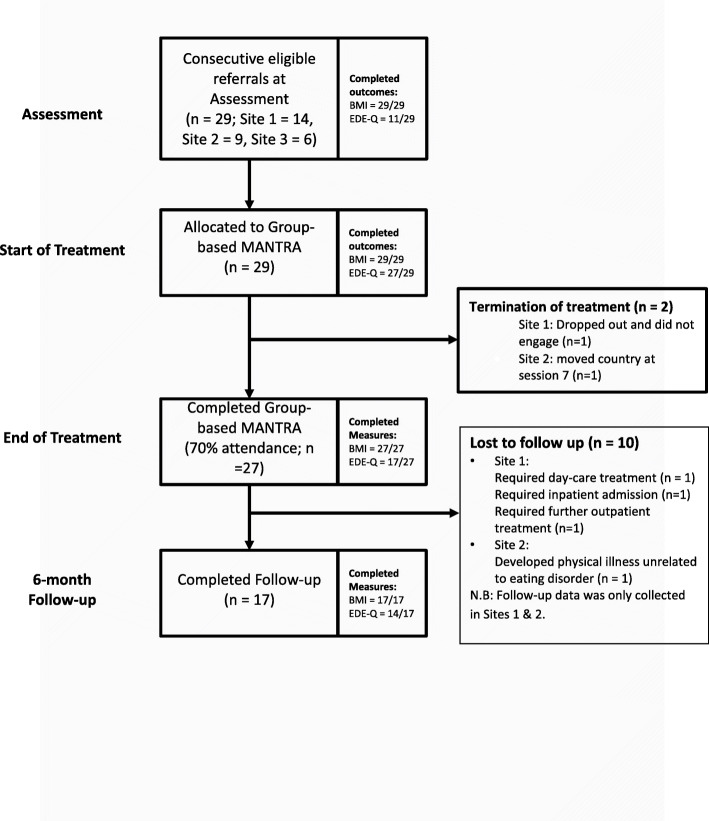
Patient flow in Group-based MANTRA

Figure 1 documents patient flow through the case series. Twenty-nine participants were recruited to Group Based MANTRA. Of those who took part in treatment, 27 (93.1%) were considered ‘completers’ by attending 70% of the programme. Participant age ranged from 18 to 54 yr (M = 29.83; SD = 10.21) and they were primarily female (89.7%). The average BMI at assessment was 16.79 (SD = 1.2) and 93.1% had a diagnosis of AN – restriction subtype.^1^ Average duration of eating disorder was 9.26 years (SD = 9.9) with mean age of onset 22.04 years (SD = 8.71). In regard treatment history; 44.8% had received previous outpatient treatment, and 13.8% a previous ED inpatient admission. Full demographics are presented in Table 2.

**Table 2:** Participant demographics, full sample (*n* = 29)

Variable	Frequency (%)
Females	26 (89.7%)
Age, *m (sd)*	29.83 (10.21)
Diagnosis	
Anorexia Nervosa – Restricting subtype	27 (93.1%)
Anorexia Nervosa – Binge/Purge subtype	2 (6.9%)
Body Mass Index (kg/m^2^), *m (sd)*	
Assessment	16.78 (1.2)
Weight (kg), *m (sd)*	
Assessment	47.26 (7.03)
Age of Onset, *m (sd)*	22.04 (8.71)
Duration of illness (years), *m (sd)*	9.26 (9.9)
Previous ED treatment	
Outpatient	13 (44.8%)
Inpatient	4 (13.8%)
Day-care	0

### Groups

In total, four groups took place across 3 sites with a median attendance of 7 participants per group session (Range = 6 to 9). Average number of group sessions attended was 15.17 (SD = 3.86) alongside 7.3 individual sessions (SD = 3.85).

### Paired samples t-test

Following completion of group-based MANTRA, there was a significant increase in BMI with a medium effect size from assessment to post treatment, *N* = 27, *p* = .013, *r* = −.293. There were insufficient data to explore EDE-Q scores at assessment, however, there was a significant decrease in EDE-Q Global score between start and end of treatment with a medium effect size, *N* = 15, *p* = .006, *r* = .366. See Table 3.

**Table 3:** Paired samples t-test for primary outcome measures

Variable	Timepoint							95% CI for Mean Difference	t	df	r
	1				2						
	M	SD	n		M	SD	n				
BMI^a^	16.78	1.21	27		17.74	1.84	27	−1.69, −.22	−2.67*	26	.293
EDE-Q^b^	3.23	1.49	15		2.15	1.24	15	.34, 1.8	3.21**	14	.366

* *p* < .05, ** *p* < .01

^a^Assessment to post-intervention; ^b^Baseline to post-intervention

### Follow up data

Where follow-up data were available (Site 1 *n* = 10, Site 2 *n* = 7) comparisons were made across all times points (assessment, baseline, post-treatment, 6-month follow-up) for BMI.

Differences in BMI scores had an average change of 1.4 (SD = 1.13) from assessment to follow-up. The full-factorial model found significant differences between mean BMI score across timepoints, F (1.65, 26.39) = 15.32, *p* < .001, MSE = .78, ηp2 = .995. Bonferroni post-hoc analysis revealed BMI to significantly increase between assessment (M = 16.91, SD = 1.20) and post-treatment (M = 17.98, SD = 1.59, CI [− 2.03, −.13], *p* < .03) and assessment and follow up (M = 18.3, SD = 1.44, CI [− 2.22, −.57], *p* < .001). Furthermore, significant differences were observed from baseline (M = 17.39, SD = 1.37, CI [− 1.15, −.03], *p* < .04) to post-treatment but there was no significant difference between assessment and baseline or post-treatment and follow-up, *p* > .05.

### Thematic analysis

Eleven participants completed five open-ended questions regarding their personal experience of the Integrated Group Treatment. Analysis is presented under five themes: 1) The group in context, 2) Bringing MANTRA concepts to life, 3) A space to be authentic, vulnerable and understood, 4) Support, empathy and care in the group setting and 5) Others as a motivator for change.

#### The group in context

Participants highlighted elements of the environmental, organisational, social and personal context in which group-based MANTRA occurred that impeded or enhanced their experience.

For example, participants tended to find the duration of the group (90 mins) acceptable; longer than 90 mins was predicted to have taxed concentration. Morning sessions were liked. Sitting on floor cushions were viewed particularly positively as they made the group feel more “informal” and as a result contributed to it being “easier to share things with the group.”

Several participants raised issues relating to the mix of people in the group; one mentioned that she was the only young person in the group and another that he was the only man. However, both felt that this did not negatively affect their experience of the group, or how supportive they found it:

*“I know all too well society’s awareness and acceptance that men also face the challenge of eating disorders is not where I would like it to be. What I can genuinely say is that the support from my therapists and the group was equal to all with gender having no bearing on the level of empathy and understanding of my situation.”*

Participants expressed challenges linked to fitting group-based MANTRA into their lives. However, many participants found the regular slot a helpful addition to their life which extended beyond the time spent in the group:

*“Having a dedicated time slot in the week to think about all this, whether I wanted to or not. It was a useful thing to hold on to in weeks which felt like I have a lot to say (“Hold on till Friday, talk it through then”) and a reminder not to ignore/pretend/forget in weeks where I was burying my head in the sand.”*

#### Bringing MANTRA concepts to life

Participants had largely positive responses to the ‘creative exercises’ used in group-based MANTRA, including ‘chairwork’, the value/identity box, using objects, music, movement, space, the body and literature to connect to emotions, letter-writing and pictorial cards. The over-arching theme in participant responses was that they were helpful in enabling participants to express difficult emotions or thoughts to the group, making the process “less daunting” particularly if they were struggling to put feelings into words:

*“I found [the Communicube] very helpful as quite often it helped me speak about things I was bottling up and express and receive support on issues troubling me.”*

Participants valued exercises where core aspects of the MANTRA programme could be realised either spatially or relationally with other group members. These included use of ‘chairwork’ as a tool for gaining a new perspective on their eating disorder, getting other group members to act out compassionate voices, and use of objects in order to stimulate reflection on values and identity. Overall participants felt that these activities were interactive and engaging and ‘helped bring the concepts to life’:

*“when talking about perspectives we actually changed our positions in the room/stood on chairs and looked at our ED thoughts from different angles to encourage us to always do this when battling with them”.*

*“There was one when we were in groups and trying to answer each other questions/be the compassionate voice the partner couldn’t hear. I remember coming away feeling a bit more settled”.*

The values box exercise was highlighted as a strong tool and there was a request that this is introduced at an early point to accompany the treatment journey. Participants were overwhelmingly positive about the course content and supporting materials, finding the structured approach helpful and valuing the workbook as a resource to look through. There was a wide-range of course components that participants rated as being most useful (with no single component standing out in analysis), suggesting that the diversity of modules was important in catering to differing needs in the group. Some suggestions to improve course content were to tie it more into the MANTRA book with specific things to read alongside each module, and to set weekly challenges.

Participants particularly valued some of the adaptations made to the MANTRA programme in order to integrate it with the group dynamic and use other group-participants to bring MANTRA concepts to life in novel and therapeutically effective ways:

*“Engaging with thinking styles and relationships with others exercise and discussing these as a group because it bought it to life and helped to apply the new strategies during the rest of the week.”*

#### A space to be authentic, vulnerable and understood

A core component of the group-based MANTRA programme valued by participants was the creation of a safe space where they were able to be open and vulnerable with others who shared a diagnosis of AN. This was felt to increase connection with their own emotions and reduce shame and isolation, however it required careful management from therapists in order to reduce the negative consequences of being ‘triggered’ by others in the group setting.

Participants consistently emphasised the therapeutic importance of being open, honest and expressing themselves in the group. Participants reported that the MANTRA groups were “safe spaces to express [your]self”, free from shame, judgment or criticism, which facilitated this process. Being vulnerable in this way not only enabled participants to feel ‘valued as an individual’ but also, through connections to others, helped them connect with their own emotions:

*“Open up as much as possible sharing vulnerabilities helps others and is an important part in connecting with emotions.”*

Significantly, as a result of listening to others’ experiences of AN and sharing their own, participants reported that they felt less ashamed about their eating disorder. Listening to others voice similar thoughts and feelings was a powerful tool for overcoming this:

*“Being able to say how I really felt and have other people: a) agree that they felt like that or thought like that; b) understood and didn’t judge me. It was reassuring to know that some of the ways I used to think (which I thought were horrible and was ashamed of) other people thought as well.”*

*“The value this brings is a feeling that maybe you’re not insane, not alone, and this is simply a challenge in life to slowly work through.”*

The challenge of ensuring the group was not damaging to participants was also recognised in their feedback. Participants had worries prior to the group commencing about it being potentially ‘triggering’, and during the group programme participants reported that listening to other’s struggles and making comparisons too often gave substance to their “ED critics”. However, alongside this, participants also recognised that engaging with others in the group was a necessary means to engage with their own emotions. Being able to discuss, reflect on and cope with the often distressing feelings that had arisen as a result of others’ contributions to the group has the potential to be an important mechanism by which group treatment for AN could be effective, if managed sensitively.

#### “We really rooted for each other”: support, empathy and care in the group setting

Participants also valued the support they received from the group, and the opportunity to support others. Within the therapy group it was felt that participants really “rooted for each other”, with a reduced sense of aloneness or isolation being the common cited helpful element of the approach. Support from other group members was particularly valued, although several participants commented on valuing “supportive and encouraging” therapists as well:

*“It’s enlightening hearing others with similar feelings and you’ll be surprised how similar they are and how support and a simple smile or understanding nod from a stranger can make the world of difference.”*

Equally, giving support and comfort to others through the process was important to participants: *“The opportunity to share our struggles & go some way to helping others has been invaluable.”*

*“Meeting new people and comforting them with my own experience”.*

#### Others as a motivator for change

Participants reported being encouraged by listening to others’ experiences of recovery. Offering a sense that change was possible, with a suggestion that it would be helpful for people who had benefited from previous groups to come back and talk about their experiences to enhance this aspect of group-based MANTRA. Furthermore, others’ accounts broadened participants’ conception of what recovery could mean, not just in terms of being better able to step away from anorexic thoughts and behaviours, but in rediscovering an authentic self, separate from the illness:

*[“Until then I had always believed I would just always think like an anorexic but just at a higher weight. A clinician could have told me I won’t have believed them because I’d think, ‘how do you know?’ ‘I’ve always thought like this, I don’t believe you that I could stop believing that (i.e. that I have to exercise every day, I can’t eat nice food because I don’t deserve to). But to hear X someone who has had anorexia and has recovered say “I used to believe xxxxxx but now I can honestly say I don’t” gave me huge encouragement. It helped me believe that it was my ED, and not me underneath, that believed that thought pattern and that there was therefore hope that the me underneath didn’t believe it.”*

Another key source of motivation was the process of empathising with others in the group stimulating self-empathy, and self kindness. This was connected to a recognition (often less possible in a solely self-directed way) that AN was something which caused huge loss of value in people’s lives. This recognition often helped participants make radical changes in their own lives:

*“It was the push I needed to give myself over to inpatient treatment as I saw what the ED was taking away from me and that it was not my friend and how it was tearing down the wonderful ladies in front of me. I couldn’t believe that they didn’t see the worth or all of their wonderful qualities.”*

## Discussion

This paper describes a feasibility case series of the integrated group-based delivery of MANTRA, where group processes and experiential techniques were used to target social and emotional processing. Quantitative and qualitative methods were used in analysis. Our primary goal explored feasibility questions including treatment retention, drop out, acceptability and preliminary treatment outcomes as delivered across three NHS sites in the UK. We were also interested in learning about what was valuable and what could be improved regarding the treatment experience.

Preliminary findings suggest that an integrated group-based MANTRA approach (Franklin-Smith, 2019) was acceptable. Of those who took part (*n* = 29), only two dropped out (7%), one who was engaged well but had to move country. Preliminary signs of effectiveness were seen, with medium effect size (pre vs post intervention) changes in regard eating disorder cognitions and behaviours (as measured by the EDEQ) and in terms of weight and BMI (from assessment to post intervention). This is encouraging given our sample of AN patients were as unwell as patients in recent large trials – [11, 12, 49], but with longer-lasting illness [50]. These findings provide preliminary feasibility data for an Integrated Group Based MANTRA approach. A randomised controlled trial (RCT) (group vs individual MANTRA) exploring postulated mechanisms of change (social and emotional processing) on core outcome (EDEQ and BMI) with a comprehensive follow up period, would enable comprehensive exploration.

The qualitative component is valuable in regard the potential of this adaptation to an already evidence-based treatment. Framework analysis methodology was used [47] to explore themes arising from semi-structured questions completed by participants at the end of treatment. The first theme: ‘The group in context’, largely validated the practical arrangements such as the length of the group, the importance of ‘creature comforts’ such as cushions, and spoke to how hard people fought to prioritise time for the treatment. As therapists securing room space was difficult. These comments remind us of the importance of the environment in enabling people to feel safe and valued.

The three remaining themes were relational and highlighted the value of the group element of the treatment and the experiential techniques. Theme two: ‘Bringing MANTRA concepts to life’, suggested that participants valued the experiential techniques to enable them to express feelings that were difficult to articulate (i.e. via the communicube), to augment perspective shifting and emotional processing (via movement in the room and chair work) and to ‘bring body and life to aspects of the self’, such as via the identity box. This theme speaks to the value of experiential methods to ‘lift’ tasks ‘off the page’ when targeting the social and emotional domain within the MANTRA treatment [30, 34, 35]. The third and fourth themes, i.e. ‘A space to be authentic, vulnerable and understood’ and ‘We really rooted for each other’, underscored the value of the group in providing a safe setting for self-expression, and to receive and offer compassion and encouragement, targeting the shame, isolation and stigma often reported by individuals with AN [51]. The fifth theme ‘Others as a motivator for change’ suggested a value in group members coming from different stages of the illness. Seeing others making changes, even after decades of being unwell, afforded a shift in perspective that enhanced personal motivation to change, creating an atmosphere of hope. Important too was the way in which offering empathy to others, prompted self-empathy. Of note, negative competitive dynamics did not take hold at any point. These were an unwell, chronic group of individuals with AN who cared for and supported each other, via effective and skilled group facilitation to empower each other and themselves to make good clinical progress. These are powerful examples of social and emotional change realised within a relational context [18, 29, 30].

This feasibility case series of an integrated group-based approach to delivering MANTRA provides preliminary indications that a group setting with an experiential focus is a useful arena for targeting emotional and social difficulties in those with Anorexia Nervosa, whilst also achieving improvements in broader outcomes. However, the freedoms and creativity of a ‘real world’ case series naturally afford some limitations. The study is underpowered, particularly in regard the follow up data, to draw conclusions about the effectiveness of the intervention or about its generalizability to specific ED presentations, such as AN binge/purge sub-types. There are a number of confounding variables that make it difficult to disentangle active ingredients of change. Of note, the study took place across three different NHS sites, with different therapists at each base and of course we describe a treatment approach that necessitated both an individual and group component. Furthermore, there was also a flexibility afforded to therapists in the treatment delivery which may hinder replication. An RCT of group vs individual MANTRA could provide a useful next step to assess AN patients’ willingness to be randomly allocated to these different treatment modalities and to use a broader range of outcome measures. Future trials should also focus on questions of cost and cost-effectiveness of using group versus individual MANTRA in AN. Clear in the feedback was the power of the group in minimising shame, loneliness and stigma around having an eating disorder. Therapists said they were honored to accompany their patients on this journey.

*“After so many years living with anorexia, I really feel now as though I am finally moving on and standing on my own” (group member).*

## Conclusion

This case series suggests that delivering MANTRA in an integrated group format is a feasible and effective intervention for individuals with severe and enduring Anorexia Nervosa. Opportunities for live social and emotional experiences within the group arena were maximized to reduce shame and encourage alternative ways of managing emotion. Future research is needed to confirm the effectiveness and mechanisms of change of this Integrated Group based approach.

## Data Availability

All data is available on request from the corresponding author on reasonable request. The group treatment manual can be requested from MFS.

## Abbreviations

AN: Anorexia Nervosa
MANTRA: Maudsley Anorexia Nervosa Treatment for Adults
WoT: Window of Emotional Tolerance

## Footnotes

1 Of available data (*n* = 27), 3 participants engaged in regular purging and 4 in regular bingeing, defined as at least once per week over the past 28 days [48].
